# Evaluation of Thermal Degradation of Tropane and Opium Alkaloids in Gluten-Free Corn Breadsticks Samples Contaminated with Stramonium Seeds and Baked with Poppy Seeds under Different Conditions

**DOI:** 10.3390/foods11152196

**Published:** 2022-07-23

**Authors:** Fernando L. Vera-Baquero, Sonia Morante-Zarcero, Isabel Sierra

**Affiliations:** Departamento de Tecnología Química y Ambiental, E.S.C.E.T., Universidad Rey Juan Carlos, C/Tulipán s/n, Móstoles, 28933 Madrid, Spain; fernando.vera@urjc.es (F.L.V.-B.); sonia.morante@urjc.es (S.M.-Z.)

**Keywords:** tropane alkaloids, opium alkaloids, corn breadsticks with poppy seeds, thermal degradation, baking process, HPLC-DAD

## Abstract

In this work, the thermal degradation of tropane and opium alkaloids was studied in samples of breadsticks prepared with corn flour, contaminated with seeds of *Datura stramonium*, and containing seeds of *Papaver somniferum* L. A total of seven different samples were prepared and eight alkaloids were studied, three tropane (atropine, scopolamine, and anisodamine) and five opium (morphine, codeine, thebaine, papaverine, and noscapine) alkaloids. For this purpose, a fast, easy and efficient method based on solid-liquid extraction (SLE) prior to the analysis by high-performance liquid chromatography with a diode array detector (HPLC-DAD) was developed and validated. Thermal degradation studies showed a decrease in the TAs and OAs content under baking (180 °C for 20 min) that was between 7–65% for atropine, depending on the preparation conditions used, between 35–49% for scopolamine and anisodamine, up to 100% for morphine and codeine and between 14–58% for thebaine, papaverine, and noscapine. Results also evidenced that degradation of morphine and codeine was higher when the seeds were added as topping to the breadsticks.

## 1. Introduction

Bakery products based on cereal flours have a long history of development and are consumed in high quantities on a daily basis, as well as playing an important role in human nutrition. Cereal flours are nutritionally important sources of macronutrients (carbohydrates, proteins) and micronutrients (some B-group vitamins, vitamin E, magnesium, zinc), as well as containing dietary fibre and bioactive compounds, so they are an essential part of a healthy diet. Bakery products that contain wheat flour as their major ingredient represent a large proportion of the cereal-based food market in most countries of the world. However, there is currently a great deal of interest in the fact that other cereals and pseudocereals are highly nutritious grains with potential applications in the production of bakery products. The growing general demand for novel, tasty and healthy foods, together with the increasing number of people suffering from wheat-related diseases, has given rise to a new market of cereal products made with grains different from wheat and rye [1]. The increasing production of wheat-free foods on the market has been a consequence of increased awareness and diagnosis of celiac disease [2], gluten sensitivity [3] and wheat allergies [4]. Buckwheat flour and other gluten-free flours (i.e., corn, rice, millet, amaranth, among others) are used as substitutes for wheat flour in the production of bread, cakes, biscuits, pasta, and snacks. These gluten-free foods have gained great popularity in the last years, especially in the celiac community [5]. In addition, one of the current trends in the preparation of bakery products is the addition of other ingredients, such as seeds. Pumpkin seeds (*Cucurbita pepo*), chia seeds (*Salvia hispanica*), sesame seeds (*Sesame indicum*), flax seeds (*Linum usitatissimum*), and poppy seeds (*Papaver somniferum*) are added to bread and other bakery products because of their high nutritional value, their health benefits in the prevention of cardiovascular diseases, their antioxidant potential and their significant contribution to dietary fibre intake [6,7,8,9,10].

Nonetheless, due to the increasing consumption of bakery products made from some gluten-free cereals and pseudocereals, it is necessary to control these ingredients, as they may be contaminated with tropane alkaloids (TAs) [11]. TAs are a remarkable class of secondary metabolites naturally occurring in numerous plants throughout the world. Atropine and scopolamine are the best-known alkaloids belonging to the tropane group and they are strong antimuscarinic agents [12,13]. Contamination with TAs can be accidental if the cereals are unintended mixed with parts of plants that naturally contain these toxic compounds, usually from the Solanaceae family. This is often due to the cereal cleaning practices, which are not always effective for weed-seeds removal. On the other hand, some seeds used in the preparation of bakery products, such as poppy seeds, have been shown to contain high amounts of opium alkaloids (OAs) [14]. OAs represent different classes of compounds isolated from *Papaver somniferum* L. This plant contains more than 40 individual OAs, the main ones being morphine, codeine, thebaine, papaverine, and noscapine, which can be used as analgesics and narcotics, cough suppressant, muscle relaxant, or as an anti-tumour agent [15]. These compounds are synthesised, stored, and metabolised in the latex (milky sap) that permeates all parts of the plant, except the seeds. Therefore, mature seeds do not contain milky sap but OAs may be present on their surface because of contamination during harvest and/or due to improper processing, as well as insect damage due to chewing of larvae on the immature capsule wall [16].

Due to the potential risk to human health from frequent ingestion of TAs and OAs contaminated bakery products, it is of utmost importance to monitor the occurrence of both toxins in foods. Accordingly, regulations to monitor the occurrence of these alkaloids in some food products have recently been published [17,18]. For OAs, the Commission Regulation (EU) 2021/2142 amending Regulation (EC) No 1881/2006 as regards the maximum level of OAs in certain foodstuffs sets 1.50 mg/kg in bakery products containing poppy seeds and/or derived products. Bakery products include also flour-based ready-to-eat savouries and snacks [17]. In the case of TAs, Commission Regulation (EU) 2021/1408 amending Regulation (EC) No 1881/2006 as regards maximum levels for TAs in certain foodstuffs establishes a maximum level of 1.0 µg/kg for atropine and 1.0 µg/kg for scopolamine in processed cereal-based foods and baby foods for infants and young children, containing millet, sorghum, buckwheat, corn or their derived products. In addition, the maximum levels for the sum of atropine and scopolamine have been set for unprocessed or processed millet, sorghum, corn, corn for popping, and buckwheat (5–15 µg/kg) [18].

Despite there being several studies focused on TAs and OAs contamination, there are only a few studies assessing how these compounds may be affected by thermal conditions during baking [19,20,21,22,23]. The European Food Safety Authority (EFSA) considers that dietary exposure to TAs and OAs should be assessed and has therefore provided some recommendations on good practices to avoid their presence during processing. It has also been highlighted the need for better characterisation of these alkaloids in food and feed, whether naturally occurring or as contaminants, as well as the recommendation of collecting analytical data on their presence in cereals and poppy seeds, considering that the raw materials in which these compounds are found are used in the production of bakery products. For these reasons, it is important to study parameters such as baking temperature and time, the quantity of seeds added, and how the seeds are incorporated into the dough (mixing them into the dough or coating the top surface). In that respect, the evaluation of these variables can help to determine the final concentration of these toxic substances in the products ingested by consumers and perform an estimation more reliable of the real intake of these alkaloids by the population. However, to our knowledge, there are no studies where TAs and OAs have been analysed simultaneously in foods, so it is important to develop simple multicomponent methods to be able to analyse both groups of alkaloids in contaminated bakery products.

Based on the above and considering that the consumption of gluten-free foods is increasing, the main objective of this work was to study the degradation of TAs and OAs in breadsticks prepared with corn flour, contaminated with stramonium seeds, and baked with poppy seeds under different conditions, in order to evaluate the effect of thermal processing on the final concentration of these alkaloids. For this purpose, a fast, easy and efficient method based on solid-liquid extraction (SLE) prior to the analysis by high-performance liquid chromatography with a diode array detector (HPLC-DAD) was developed and validated to simultaneously quantify three TAs and five OAs (atropine, scopolamine, anisodamine, morphine, codeine, thebaine, papaverine, and noscapine) in these samples.

## 2. Materials and Methods

### 2.1. Reagents and Materials

Anisodamine hydrobromide (racemic mixture of diastereomers), scopolamine hydrobromide and atropine were purchased from Sigma-Aldrich (St. Louis, MO, USA). Morphine, codeine, and thebaine were purchased from Alcaliber S.A.U. (Madrid, Spain). Papaverine and noscapine were purchased from Sigma-Aldrich (Zwijndrecht, The Netherlands). Stock solutions (1000 mg/mL) were prepared in methanol. A standard working solution containing a mixture of TAs and OAs at the desired concentration was prepared in water with 0.1% trifluoroacetic acid. All solutions were stored at −20 °C. Acetonitrile, triethylamine, and trifluoroacetic acid from Fisher Scientific (Madrid, Spain) and ammonium acetate from Scharlab (Barcelona, Spain) were used for HPLC mobile phase preparation. Glacial acetic acid from Scharlab, methanol, and formic acid from Fisher Scientific were used for the extraction of the analytes, all of HPLC-MS grade. Ultra-pure water (resistance 18.2 MΩ cm) was obtained from a Millipore Milli-Q-System (Billerica, MA, USA). 0.45 µm nylon membrane filters, 0.45 µm nylon syringe filters, and 1 mL empty syringes were purchased from Scharlab (Barcelona, Spain).

### 2.2. Poppy Seeds, Corn Flour, and Dough Contamination

**Figure 1:**
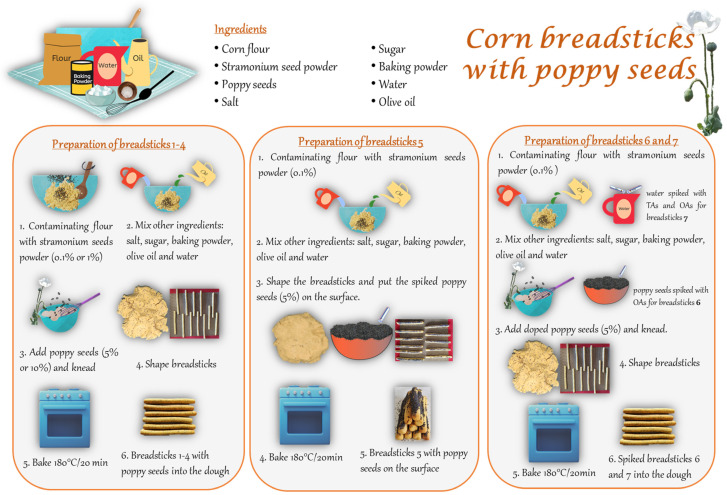
Schematic representation of the breadsticks preparation (samples 1–7).

Organic poppy seeds and organic corn flour purchased in a local supermarket in Madrid (Spain) were used to prepare the breadsticks samples as indicated in Section 2.3 (see Figure 1 and Table 1). The poppy seeds were analysed following the methodology described in Section 2.4. To evaluate the degradation of OAs that were not naturally found in the poppy seeds, the seeds (5 g) or the dough (180 g) were fortified before use with a mixture containing 60 µg of codeine, thebaine, papaverine, and noscapine. When the standards were added to the seeds, the mixture was homogenized and allowed to dry before use. When the standards were added to the dough, they were first mixed with the water used as ingredient (50 mL), in order to ensure a homogeneous distribution of the alkaloids. On the other hand, to evaluate the degradation of TAs, the commercial corn flour free of alkaloids (blank sample) was mixed with stramonium seed powder in different proportions. For this, *Datura stramonium* seeds were collected in Cáceres (Spain), ground into a fine powder, and then analysed following the methodology described in Section 2.4. To evaluate the degradation of TAs that were not naturally present in the stramonium seed powder, the dough (180 g) was fortified with 190 µg of anisodamine and scopolamine, which were added to the water.

### 2.3. Preparation of Breadsticks Samples

The dough for breadsticks was prepared by mixing 100 g of corn flour, 2.5 g of baking powder (disodium diphosphate, sodium bicarbonate and sodium carbonate), 2.5 g of salt, 2.5 g of sugar, 22.5 g of olive oil and 50 mL of water (dough weight: 180 g). Next, the dough was divided into 12 portions of 15 ± 1 g. Each piece was moulded into a 13 × 0.5 cm (length × width) rope and was baked for 20 min in a Candy Fan Assisted electric oven (Madrid, Spain) at 180 °C. To measure the temperature, a T-type probe coupled to a TESTO model 926 digital thermometer (Madrid, Spain) was inserted into the center of the breadsticks, which was 99.6 °C when baking was completed (after 20 min). The breadsticks were then allowed to cool at room temperature (breadsticks weight after baking: 11 ± 1 g; weight loss: 26 ± 1%, *n* = 12). For thermal degradation studies of alkaloids, samples of breadsticks were prepared with corn flour fortified with 0.1% (0.1 g) or 1% (1 g) of stramonium seed powder, to which 5% (5 g) or 10% (10 g) of poppy seeds were added to the dough (breadsticks samples 1 to 4, see Figure 1 and Table 1). In some other preparations, the dough (180 g) or the seeds (5 g) were doped with a standard mixture of TAs and/or OAs, and the poppy seeds were added inside the dough or on the breadstick surface, to simulate different commercial bakery products (breadsticks samples 5 to 7). Figure 1 shows the schematic process for breadsticks samples preparation and Table 1 collets details for the preparation of breadstick samples 1 to 7. Samples were stored in the dark in room Tª until analysis.

### 2.4. Analytical Methodology

#### 2.4.1. Chromatographic Conditions

TAs and OAs were separated and identified using an Agilent 1260 Infinity II HPLC system (Agilent Technologies, Madrid, Spain), equipped with a flexible pump (G7104C 1260 Flexible Pump), automatic multi-injector (G7167A 1260 Multisampler), thermostated column compartment (G7116A 1260 MCT) and diode array detector (G7117C 1260 DAD HS). The Agilent OpenLab CDS ChemStation Edition software was used for data handling. Separation was achieved using an InfinityLab Poroshell 120 EC-C18 column (3.0 mm i.d. × 150 mm, 2.7 µm particle size) equipped with an InfinityLab Poroshell 120 EC-C18 guard column (3.0 mm i.d., 2.7 µm particle size) both from Agilent Technologies (Madrid, Spain). Parameters such as composition and gradient of the mobile phase, flow rate, injection volume, and wavelength were optimised. A mixture of solvent A (Milli-Q water with 0.1% trifluoroacetic acid) and solvent B (acetonitrile) was used as the mobile phase in gradient elution mode as follows: 0–2 min 2% solvent B, 2–5 min 15% solvent B, 5–6.50 min 25% solvent B, 6.50–8.50 min 35% solvent B, 8.50–10 min 35% solvent B, 10–10.50 min 2% solvent B and 10.50–12 min 2% solvent B (Table S1). The flow rate was 1.000 mL/min and an injection volume of 20 µL. The column temperature was maintained at 30 °C and the autosampler tray was kept at 4 °C. The total analysis time was 12 min. Detection was performed at 212 nm (morphine, codeine, anisodamine, scopolamine, atropine, and noscapine), 225 nm (thebaine), and 240 nm (papaverine). Figure S1 shows the chemical structures of the TAs and OAs studied in this work.

#### 2.4.2. Extraction of TAs and OAs in Breadsticks

**Figure 2:**
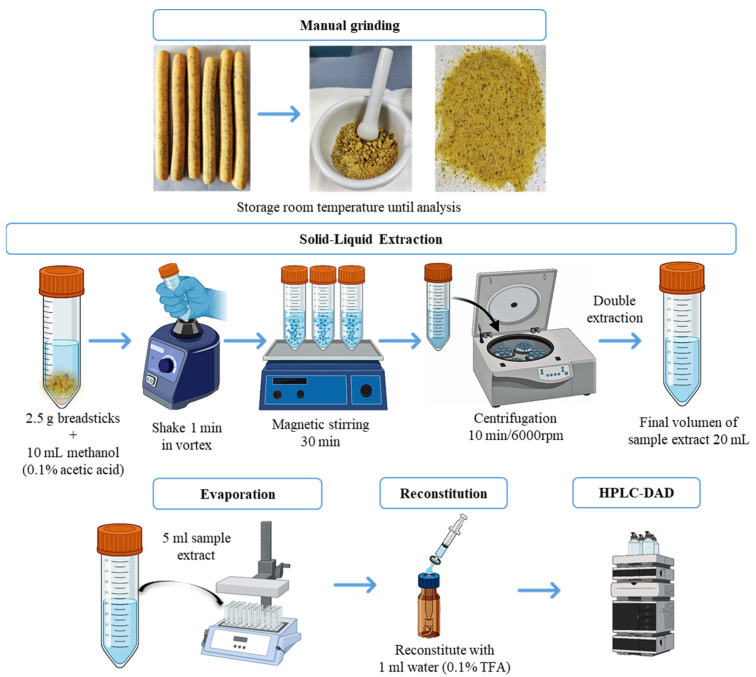
Schematic representation of sample treatment for tropane and opium alkaloids analysed in the breadstick samples.

For the extraction of the target analytes, an SLE process was developed. The following parameters were optimized: type of solvent (methanol/water, 2/1, *v*/*v*; acetonitrile/water, 80/20, *v/v*; methanol with 0.1% acetic acid) and sample amount (1.0 and 2.5 g). Under the optimized conditions, the SLE protocol was as follows (Figure 2): 2.5 g of sample (breadsticks manually ground to a fine powder) were extracted with 10 mL of methanol with 0.1% acetic acid. The sample was shaken for 1 min with vortex (Rx^3^ Velp Scientifica, Usmate, MB, Italy), 30 min with magnetic stirring (IKA RCT basic, Staufen, Germany), and, finally, it was centrifuged for 10 min at 6000 rpm (ROTOFIX 32A Hettich, Tuttlingen, Germany). Subsequently, the supernatant was recovered and a second extraction was performed under the same conditions. The two supernatants were mixed and 5 mL were taken to dryness and reconstituted with 1 mL of water with 0.1% trifluoroacetic acid to be injected into the HPLC-DAD system. Each sample was analyzed six times (*n* = 6). Results were calculated as mean concentration with its corresponding standard deviation, except for morphine because its distribution in the seeds is not homogeneous so the results are shown as intervals.

### 2.5. Method Validation

The method was evaluated in terms of linearity, matrix effect (ME), accuracy, precision, selectivity, method detection (MDL) and quantification (MQL) limits. As there is no official regulation, the method validation was performed following the criteria described in the document SANTE/11312/2021 establishing the analytical quality control and method validation procedures for pesticide residues analysis in food and feed [24]. The applicability of the method was demonstrated by the analysis of real samples of corn breadsticks with poppy seeds prepared in the laboratory and contaminated with TAs and OAs.

## 3. Results

### 3.1. Optimization of HPLC-DAD Analysis

**Figure 3:**
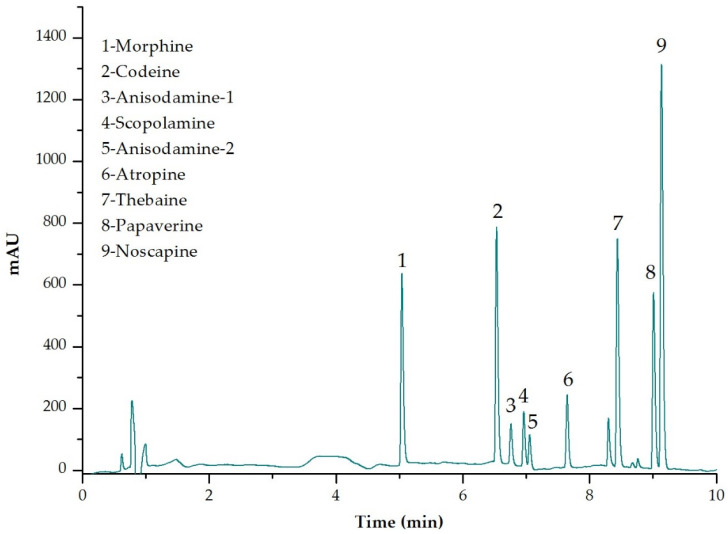
Chromatographic separation obtained for tropane and opium alkaloids under optimized conditions with the HPLC-DAD method at 212 nm.

So far, both TAs and OAs have been analysed separately. Then, the first challenge in this work was to achieve a good simultaneous separation of these two families of compounds with different characteristics. The chromatographic conditions were optimized for the simultaneous quantification of three TAs (anisodamine, scopolamine, and atropine) and five OAs (morphine, codeine, thebaine, papaverine, and noscapine). In the case of anisodamine, as the standard it is a racemic mixture of diastereomers, in the optimum conditions, two peaks were obtained, denoted as anisodamine-1 and anisodamine-2 (Figure 3). The column temperature was set at 30 °C, according to the work of Acevska et al. [25], who evaluate the effect of column temperature on the chromatographic separation efficiency of OAs. The optimum absorption wavelength (λ) was also evaluated and, finally, set at 212 nm for morphine, codeine, anisodamine, scopolamine, atropine, and noscapine, 225 nm for thebaine and 240 nm for papaverine, to obtain the maximum sensibility of the method.

Consequently, to separate the nine alkaloids, different mobile phases were evaluated in this work. In order to obtain good separation (resolution, Rs ≥ 1.5) and peak shape (peak asymmetry factor, As = 1), the effect of the formic acid [11,14,26,27] or trifluoroacetic acid [25,28] concentration in the mobile phase was evaluated. The concentration of both acids was tested in the range of 0.05 to 0.2% (*v*/*v*). Results showed that the concentration of these acids had little influence on the retention of the compounds. However, when formic acid was used there was an increase in the baseline noise at the lowest λ, which hindered the quantification of the compounds. On the other hand, with trifluoroacetic acid, the chromatographic profile improved, as well as the shape of the peaks. Triethylamine [25,26,28,29,30,31] and ammonium acetate [29,32] were also added to the mobile phase but did not improve the separation of the target compounds. Taking these results into account, a mixture of water with 0.1% trifluoroacetic acid was chosen as eluent A. To improve peak shape and reduce retention time (t_R_), the effect of acetonitrile [27,33,34,35,36] and methanol [25,26,28,29,32] as organic modifiers in the mobile phase (eluent B) was evaluated. Using the same chromatographic conditions (flow rate: 0.8 mL/min; column temperature 30 °C; elution gradient: 0–1 min 2% solvent B, 1–10 min 90% solvent B, 10–12 min 2% solvent B, 12–15 min 2% solvent B), it was observed that acetonitrile reduced the t_R_ of the compounds, compared to methanol, and also gave higher Rs. Therefore, acetonitrile was chosen as eluent B. Under these conditions, the peaks were narrower and the separation was improved. Subsequently, the flow rate and injection volume were optimized. Injection volumes from 10 to 50 µL and flow rates from 0.500 to 1.000 mL/min were tested, and 1.000 mL/min and 20 µL were selected as the best ones. On the other hand, different solvents were tested for the injection of the compounds (water, water with 0.1% trifluoroacetic acid, acetonitrile, and methanol). It was concluded that a mixture of water with 0.1% trifluoroacetic acid gave a higher Rs.

Finally, with the optimal eluents, different gradients were tested to improve the separation of the analytes. The best gradient was: 0–2 min 2% solvent B, 2–5 min 15% solvent B, 5–6.50 min 25% solvent B, 6.50–8.50 min 35% solvent B, 8.50–10 min 35% solvent B, 10–10.50 min 2% solvent B and 10.50–12 min 2% solvent B. Figure 3 shows a typical chromatogram with all compounds resolved in 12 min (λ = 212 nm). Figure S2 shows the chromatograms of each analyte at the λ of its maximum absorption under the optimized chromatographic conditions. As can be seen in Table S2, t_R_ were: morphine, 5.068 min; codeine, 6.561 min; anisodamine-1, 6.796 min; scopolamine, 6.997 min; anisodamine-2, 7.085 min; atropine, 7.678 min; thebaine, 8.486 min; papaverine, 9.058 min and noscapine, 9.192 min.

The efficiency of the column was determined by the number of theoretical plates (N) according to USP. The value estimated with the last peak in the chromatogram (noscapine) was near 220,000 (0.075 mm/plate) which indicates a good column efficiency. As can be seen in Table S2, the retention factor (k) was between 5.3–10.4, and the separation factor (α) was between 1.0–1.2. and Rs was always ≥1.4, with an As between 1 and 1.19.

### 3.2. Optimization of the Extraction of TAs and OAs in Breadsticks

Considering the study of Lachenmeier et al. [19] that reported that grinding poppy seeds reduces the morphine content by 25–34%, in our study manual grinding of the samples (in a mortar without gridding the poppy seeds) was carried out before the extraction process. This reduction is attributed to the fact that morphine degradation is accelerated in the presence of oxygen, and that grinding provides a larger and more active surface area for reaction with it, resulting in the formation of pseudomorphine and morphine-N-oxide [21].

A review of the extraction protocols in the literature shows that methanol [25], acidified methanol [21], acidified water [37,38], and a mixture of solvents [14,20,39,40] are used to extract OAs from both poppy seeds and foodstuffs. TAs can be extracted from foods using acidified water [11] or a mixture of solvents [22,41,42]. Based on these previous papers, three types of extraction solvents were evaluated in our study: methanol/water (2/1, *v*/*v*) with 0.5% acetic acid, acetonitrile/water (80/20, *v*/*v*) with 0.1% formic acid and methanol with 0.1% acetic acid. In addition, according to the study of Casado-Hidalgo et al. [14] two successive extractions with 10 mL of solvent were considered sufficient to extract the compounds of interest. Breadsticks samples prepared with TAs-free corn flour containing OAs-free chia seeds were used in order to avoid the presence of both types of toxic alkaloids in the sample. In these experiments, 1 g of sample was subjected to the extraction process. The samples were spiked before the extraction process with 100 µL of a standard TAs and OAs solution of 100 ppm and compared with a simulated sample that was spiked after the extraction process and before evaporation. The recovery was estimated by comparing the areas of the spiked and simulated samples. Figure S3a shows the recoveries (%) obtained with the three solvents. As it can be seen, with the exception of thebaine, low recoveries (lower than 25%) were obtained with methanol/water (2/1, *v*/*v*) with 0.5% acetic acid as extraction solvent. On the other hand, with acetonitrile/water (80/20 *v*/*v*) with 0.1% formic acid and methanol with 0.1% acetic acid, recoveries above 90% were obtained. According to these results, methanol with 0.1% acetic acid was selected as the extraction solvent for the next experiments, not only because of the good recoveries achieved and low RSD values, but because this solvent is easy to evaporate prior to the chromatographic analysis. Subsequently, taking into account the low concentrations in which the alkaloids will be found in the samples, the amount of samples to be used in the experiments was evaluated and 2.5 g of samples were tested. Good recoveries of 93% to 99% and RSD values < 5% were obtained (Figure S3b). Larger amounts of the sample were not tested not to increase the volume of solvent extraction. Consequently, 2.5 g of sample amount was selected.

### 3.3. Method Validation

The reliability of the developed method was carried out by evaluating parameters such as linearity, accuracy, precision, selectivity, LOD, LOQ, and matrix effect (ME), following the recommendations described in SANTE/11312/2021 [24]. Data for the instrumental validation of the HPLC-DAD analysis are included in Table S3 and the validation parameters of the method are shown in Table 2. Method linearity was assessed with matrix-matched calibration curves. For this purpose, the sample extracts obtained after the SLE procedure were spiked with an aliquot of a standard solution containing TAs and OAs according to the desired concentration level of the calibration curve. A regression analysis of the area of each peak versus concentration was used, obtaining in general coefficients of determination (R^2^) > 0.999. The method was linear in the range of 0.2–6.0 mg/L for morphine, scopolamine, thebaine, papaverine and noscapine, 0.2–3.0 mg/L for codeine, 0.6–6.0 mg/L for anisodamine-1, 1.0–6.0 mg/L for anisodamine-2 and 1.0–10 mg/L for atropine. MDL and MQL were estimated as three and ten times, respectively, the S/N for the chromatographic response in HPLC-DAD to the lowest concentration in the matrix calibration. MDL and MQL were between 0.03–0.32 mg/L and 0.12–1.02 mg/L, respectively (Table 2). The selectivity of the method was assessed by checking the DAD spectra, retention, time, and purity of the peaks obtained for the analytes from the breadsticks samples, compared with standard solutions. To evaluate the ME, samples of breadsticks made with corn flour were used. ME was calculated by comparing the slopes of matrix-matched calibration curves with solvent-based standard calibration curves by means of the following equation: (matrix slope/solvent slope) × 100. The ME obtained ranged between 80 and 109%, so none of the compounds were affected by the ME after extraction. The accuracy of the method was established as the average recovery of six samples (*n* = 6) spiked with the analytes at known concentrations, after which the extraction process described in Section 2.4.2 was applied. The concentration levels at which the accuracy was evaluated were: low (0.08 mg/kg for OAs and 0.30 mg/kg for TAs), medium (3.0 mg/kg for TAs and OAs) and high (6.25 mg/kg for TAs and OAs). The recovery values were calculated by comparing the values of samples spiked before the extraction process with the simulated samples that were spiked after the extraction process and before evaporation. The values obtained ranged from 96 to 110% (Table 2). Finally, intra-day and inter-day precision expressed in RSD (%), showed values below 18% and 16%, respectively. All validation parameters were according to the criteria established in the SANTE/11312/2021 [24]. The low standard deviation values showed that repeatability and precision were very good. The results obtained demonstrated that the proposed method showed good analytical performance and can be successfully used for the extraction and quantification of TAs and OAs in real samples.

### 3.4. Evaluation of Thermal Degradation of TAs and OAs in Baked Breadsticks

#### 3.4.1. Reduction of Alkaloids Naturally Present in the Stramonium and Poppy Seeds

Breadstick samples 1–4 were prepared using fortified corn flour and poppy seeds (see Figure 1 and Table 1) to study the degradation of the alkaloids that naturally occur in the stramonium and poppy seeds used. For this purpose, the corn flour was previously mixed with stramonium seed powder and different proportions: 0.1% (breadsticks samples 1 and 2) or 1% (breadsticks samples 3 and 4). Firstly, the stramonium seed powder was analysed with the proposed method, and the atropine concentration was found to be 1911 ± 283 mg/kg (*n* = 6). This amount was in accordance with other previous studies [43]. Scopolamine and anisodamine were not detected in the stramonium seeds analysed. On the other hand, different proportions of organic poppy seeds were added to the dough: 5% (breadsticks samples 1 and 3) or 10% (breadsticks samples 2 and 4). In these seeds, morphine was found to be between 0.23–3.72 mg/kg (*n* = 12). Results are given in ranges because the concentration of OAs in seeds is not homogeneous as the presence of these compounds is due to accidental contamination, which makes the concentration variable even in the same batch of seeds. These concentration values are in agreement with those determined by other researchers (see Table S4). Other OAs (codeine, thebaine, papaverine, and noscapine) were not detected in the poppy seeds analysed. For this reason, experiments with breadstick samples 1 to 4 were carried out to evaluate the degradation of TAs (atropine) and OAs (morphine) naturally found in the poppy and stramonium seeds.

Table 3 shows the concentration of morphine and atropine found in the breadsticks samples 1–4, after baking at 180 °C for 20 min. Table S5 (see Supplementary Information) shows the expected concentration of the target analytes in the breadsticks before and after baking and the concentration found in the analysed baked breadsticks samples. As can be seen, an important reduction of atropine and morphine in relation to the initial content of both compounds in the dough was found. In the case of atropine, after baking a concentration of 0.12–0.14 and 0.51 mg per 100 g sample (dry weigh, d.w.) was found in the breadsticks which means a reduction of 20% in sample 1, 7% in sample 2 and 64–65% in samples 3 and 4. These results indicated that the higher amount of stramonium seed powder in the dough favored a higher thermal degradation. On the other hand, a reduction of morphine up to 27% was also observed, but despite this degradation, appreciable amounts of morphine between 0.012 and 0.036 mg per 100 g sample remained in the breadsticks (Table 3).

#### 3.4.2. Reduction of Alkaloids in Samples Spiked with Standards

Table 4 shows the concentration of TAs and OAs in the spiked breadsticks samples 5–7 (see Figure 1 and Table 1). Firstly, to see the influence of the temperature on the degradation of the target OAs, an assay was carried out in which poppy seeds were applied to the surface of each breadstick (breadstick sample 5). In this sample, 0.1% of stramonium seed powder and 5% of poppy seeds were used. To estimate the thermal degradation of the OAs that were not naturally found in the poppy seed, they were spiked with a standard solution containing 60 µg of codeine, thebaine, papaverine, and noscapine. As can be seen in Table 4, when these breadsticks were analysed, some of the OAs could not be quantified because they were below the MQL of the method, as was the case of thebaine, although a reduction > 58% was estimated for this alkaloid. In the case of morphine and codeine, the presence of these two compounds could not be detected after the baking process, which means a reduction of almost 100%. For papaverine and noscapine, the reduction estimated was 55% and 23%, respectively (Table 4). In the case of TAs, the atropine was reduced by up to 35% after baking. Comparing these results with those obtained in the breadstick sample 6 (similar preparation to sample 5 but with the doped poppy seeds inside the dough), it could be concluded that the degradation of morphine and codeine was higher when the seeds are on the surface of the breadsticks than when they are inside the dough. This fact indicates that the matrix plays an important role in protecting both compounds from thermal degradation during baking, as the temperature inside the dough was 99.6 °C and on the surface 180 °C. However, for thebaine, papaverine, and noscapine no significant differences in the reduction between samples 5 and 6 were found.

Finally, to estimate the degradation of TAs that were not naturally found in the stramonium seed powder, breadstick sample 7 was prepared with fortified corn flour (with 0.1% of powdered stramonium seeds) and 5% of poppy seeds added into the dough. This sample was spiked with a standard solution containing 60 µg of codeine, thebaine, papaverine, and noscapine and 190 µg of anisodamine and scopolamine as indicated in Section 2.3 (see Figure 1 and Table 1). Results (Table 4) show that after baking, morphine concentrations of 0.003–0.007 mg per 100 g sample are found, resulting in a reduction of up to 77%. In the case of codeine and thebaine, the concentration was below the MQL, although a reduction > 88% for codeine and >58% for thebaine was estimated. Despite the evidenced thermal degradation, appreciable amounts of codeine and thebaine remained in the sample, 0.004 and 0.003 mg per 100 g, respectively. Under the same processing conditions, papaverine was reduced by 49%, while noscapine was reduced by 14%. On the other hand, there was also a significant reduction in the TAs after the baking process. According to other assays, atropine was reduced by up to 32%, although appreciable concentrations of 0.11 mg per 100 g of the sample remained. Higher reductions were observed for scopolamine, around 45%, whereas for anisodamine-1 and 2 lower reductions (35 and 49%, respectively) were observed (Table 4).

## 4. Discussion

There are few references in the literature evaluating the degradation of TAs and OAs due to thermal cooking processes and, to the best of our knowledge, there are no studies where both groups of the toxic compounds are evaluated simultaneously. This is an important issue due to the concentration of TAs and OAs can be modified during cooking conditions, as well as, other compounds can be generated [44]. An overview of TAs and OAs reduction during thermal processing of different cereal-based foods is shown in Table S6.

In the case of TAs, there are few studies evaluating the effect of thermal processing on these compounds. In the work by Marín-Sáez et al. [22] the influence of proofing and baking on the degradation of TAs in bread made with buckwheat flour contaminated with Solanaceae seeds (*Datura stramonium* and *Brugmansia arborea*) was evaluated. The study showed that after proofing (37 °C, 60 min) there was a reduction of 35, 54, and 34% in anisodamine, scopolamine, and atropine, respectively. However, after subsequent baking (190 °C, 40 min) this reduction was higher: 83% for anisodamine, 84% for scopolamine, and 73% for atropine. These percentages of degradation are higher than those observed in our study (in the range of 7–65%) and can be attributed to the absence of the fermentation step in the breadstick preparation. In addition, these authors observed that the degradation of TAs was lower in the “real samples” than in vial trials, which was explained due to the extra protection of the matrix against compounds. This fact is consistent with the results observed in our study, because a higher reduction in the atropine content was observed in samples with a higher content of stramonium seed powder (around 65% in samples 3 and 4). In any case, the final concentration of atropine and scopolamine in the baked breadsticks was significantly lower, so it can be said that this thermal process is an effective way to reduce contamination with these toxic compounds. On the other hand, in other types of cereal-based foods, such as pasta, Marín-Sáez et al. [23] evaluated how the boiling process affects these compounds and whether they degrade or migrate to the aqueous phase. Their results show that during the processing of pasta samples contaminated with *D. stramonium*, atropine and scopolamine degraded around 60%, while anisodamine degraded around 40% (Table S6). Comparing the concentration of TAs detected in both phases (paste and boiled water), it was observed that most of the compounds were mainly degraded or migrated to the water (around 20–40%). For this reason, these authors concluded that treatment with hot water (100 °C) can be an effective way to decontaminate samples such as other cereals, pseudocereals, or processed foods.

Up to now, only a few studies evaluated changes in levels of OAs during thermal processing. As it can be seen in Table S6, morphine and partly codeine were the main subjects of degradation studies. Nevertheless, the consumption of other OAs (for example thebaine) may also have adverse health consequences in humans [45]. On the other hand, dependent upon the food matrix (muffin, buns, cake, bread) and heating conditions, ambivalent results were reported, ranging from a nearly full OAs degradation to hardly any degradation. These results can also be explained by the fact that variability in the content of OAs within different proportions of the same batch can be high [14], and this makes it difficult to adequately evaluate the reduction after thermal treatments [46]. In this sense, Kleinmeier et al. [46] and Kuntz et al. [47] point out this controversial issue in the scientific literature. For example, Shetge et al. [20] found that morphine, codeine, and thebaine were relatively stable in muffins after 16 min of baking at 200 °C. Similarly, no reduction in opium alkaloid concentrations was observed when the poppy seeds were applied to the muffin surface. On the contrary, Sproll et al. [21] evaluated the effect of food processing on morphine and codeine content. Their results show a significant reduction in morphine and codeine. For poppy cake (180 °C, 20 min) a 50–84% reduction of morphine and 50–90% of codeine was found. When poppy seeds were used as a baking topping (for poppy buns) at the highest temperature (220 °C) a reduction of 97 and 93% for morphine and codeine, respectively, was observed. Reductions near 100% were also observed for morphine, codeine, papaverine, and noscapine in muffins baked at 180 °C for 15 min and in poppy seeds topped rolls (190 °C, 25 min) by Carlin et al. [40]. Additionally, Kuntz et. al. [47] showed that the highest recovery of morphine in cakes prepared with unground seeds was 50% (180 °C, 20 min), while the lowest recovery for cakes with ground seeds was 16%. When comparing the results from the current work to the reductions reported in other words, it can be seen that these findings are in-keeping with those that propose thermal heat treatment of poppy seeds as an effective processing method to reduce the overall OAs content. In addition, it was demonstrated that the degradation of morphine and codeine is higher when the seeds are added as topping to the breadsticks. Because only a temperature of near 100 °C was recorded in the breadsticks core, the stability of OAs in these samples was assumed because of insufficient heat exposure due to matrix protection.

## 5. Conclusions

The effect of thermal processing on samples of gluten-free breadsticks prepared with corn flour and poppy seeds, and intentionally contaminated with *Datura stramonium* seeds, has been evaluated. The results obtained showed that in the baking process, the degradation of tropane alkaloids can be up to 65%. Opium alkaloids can be degraded up to 88% when the seeds are incorporated into the dough. On the other hand, when the seeds are applied to the breadsticks’ surface, an almost complete degradation of morphine and codeine was observed. Finally, as only very limited data are available, further studies addressing this topic are necessary soon, especially in less studied toxic potential alkaloids, such as thebaine.

## Figures and Tables

**Table 1:** Details for the preparation of breadsticks samples analysed in the work.

Samples	Corn Flour Amount (g)	Stramonium Seed Powder (g)	TAs ^h^ Added to the Dough (µg)	Poppy Seeds into Dough (g)	Poppy Seed on the Surface (g)	OAs ^i^ Added to the Seeds (µg)	OAs ^i^ Added to the Dough (µg)
Breadsticks 1 ^a^	100	0.1	-	5	-	-	-
Breadsticks 2 ^b^	100	0.1	-	10	-	-	-
Breadsticks 3 ^c^	100	1	-	5	-	-	-
Breadsticks 4 ^d^	100	1	-	10	-	-	-
Breadsticks 5 ^e^	100	0.1	-	-	5	60	-
Breadsticks 6 ^f^	100	0.1	-	5	-	60	-
Breadsticks 7 ^g^	100	0.1	190	5	-	-	60

^a^ Dough prepared with corn flour fortified with stramonium seed powder (0.1%) and poppy seeds into de dough (5%); ^b^ Dough prepared with corn flour fortified with stramonium seed powder (0.1%) and poppy seeds into de dough (10%); ^c^ Dough prepared with corn flour fortified with stramonium seed powder (1%) and poppy seeds into de dough (5%); ^d^ Dough prepared with corn flour fortified with stramonium seed powder (1%) and poppy seeds into de dough (10%); ^e^ Dough prepared with corn flour fortified with stramonium (0.1%) and poppy seeds (5%), added on the surface, doped with 60 µg of Cod, The, Pap and Nos; ^f^ Dough prepared with corn flour fortified with stramonium (0.1%) and poppy seeds (5%), added into the dough, doped with 60 µg of Cod, The, Pap and Nos; ^g^ Dough doped with TAs and OAs (added in the water) prepared with corn flour fortified with stramonium (0.1%) and poppy seeds (5%) into the dough; ^h^ TAs: Tropane alkaloids; ^i^ OAs: Opium alkaloids, Cod: codeine, The: thebaine, Pap: papaverine, Nos: noscapine.

**Table 2:** Validation parameters of the SLE-HPLC-DAD method in breadsticks samples.

									Accuracy ^c^		Precision ^c^	
Compound	Linear Range		Matrix Calibration (mg/L), R^2^	MDL ^a^		MQL ^a^		Matrix Effect (%) ^b^	Recovery (% ± SD)	Mean Recovery (% ± SD)	Intra-Day Presicion (RSD%)	Inter-Day Presicion (RSD%)
	mg/L	mg/kg		mg/L	mg/kg	mg/L	mg/kg					
Morphine	0.2–6.0	1.6–48	y = 318.55x − 2.7892 0.999	0.05	0.40	0.17	1.36	96	95 ± 2 *^a^*	101 ± 4	2 *^a^*	7 *^a^*
									102 ± 1 *^b^*		1 *^b^*	1 *^b^*
									107 ± 11 *^c^*		10 *^c^*	6 *^c^*
Codeine	0.2–3.0	1.6–24	y = 388.9x + 0.4358 0.9996	0.06	0.48	0.21	1.68	95	97 ± 5 *^a^*	103 ± 6	6 *^a^*	5 *^a^*
									103 ± 1 *^b^*		1 *^b^*	2 *^b^*
									109 ± 12 *^c^*		11 *^c^*	7 *^c^*
Anisodamine-1	0.6–6.0	4.8–48	y = 68.925x − 3.7223 0.9999	0.18	1.44	0.60	4.80	109	99 ± 2 *^a^*	98 ± 4	2 *^a^*	4 *^a^*
									95 ± 2 *^b^*		2 *^b^*	3 *^b^*
									101 ± 8 *^c^*		8 *^c^*	2 *^c^*
Scopolamine	0.2–6.0	1.6–48	y = 78.934x − 6.926 0.9999	0.07	0.56	0.20	1.60	101	99 ± 5 *^a^*	104 ± 5	5 *^a^*	5 *^a^*
									113 ± 5 *^b^*		4 *^b^*	5 *^b^*
									102 ± 7 *^c^*		7 *^c^*	2 *^c^*
Anisodamine-2	1.0–6.0	8–48	y = 45.16x + 2.1179 0.9997	0.31	2.48	1.02	8.16	98	111 ± 2 *^a^*	110 ± 8	2 *^a^*	6 *^a^*
									101 ± 1 *^b^*		1 *^b^*	1 *^b^*
									118 ± 21 *^c^*		18 *^c^*	11 *^c^*
Atropine	1.0–10	8–80	y = 105.07x − 6.4501 0.9991	0.32	2.56	1.00	8.00	88	97 ± 2 *^a^*	100 ± 2	2 *^a^*	2 *^a^*
									105 ± 1 *^b^*		1 *^b^*	3 *^b^*
									100 ± 4 *^c^*		4 *^c^*	2 *^c^*
Thebaine	0.2–6.0	1.6–48	y = 270.86x + 5.9803 (0.9999)	0.05	0.45	0.18	1.49	85	87 ± 11 *^a^*	96 ± 5	11 *^a^*	10 *^a^*
									106 ± 3 *^b^*		2 *^b^*	6 *^b^*
									97 ± 3 *^c^*		3 *^c^*	1 *^c^*
Papaverine	0.2–6.0	1.6–48	y = 506.88x − 8.0069 (0.9997)	0.04	0.37	0.15	1.25	80	98 ± 14 *^a^*	101 ± 6	15 *^a^*	7 *^a^*
									108 ± 4 *^b^*		4 *^b^*	7 *^b^*
									98 ± 2 *^c^*		2 *^c^*	2 *^c^*
Noscapine	0.2–6.0	1.6–48	y = 634.82x − 68.207 (0.999)	0.03	0.30	0.12	1.01	82	90 ± 14 *^a^*	100 ± 7	16 *^a^*	16 *^a^*
									108 ± 1 *^b^*		1 *^b^*	4 *^b^*
									103 ± 6 *^c^*		6 *^c^*	2 *^c^*

^a^ MDL: method detection limit and MQL: method quantification limit estimated as 3 and 10 times the signal/noise ratio, respectively. ^b^ Matrix effect calculated by (matrix slope/solvent slope) × 100. ^c^ Accuracy (mean recovery obtained from six samples, *n* = 6) and precision were obtained by spiking samples at three known concentration levels. Intra-day precision: six consecutive injections (*n* = 6) on the same day; Inter-da precision: three replicate samples injected in triplicate throughout three different days (*n* = 9). *^a^* Low spiked level (0.08 mg/kg: morphine, codeine, thebaine, papaverine and noscapine; 0.3 mg/kg: anisodamine-1,-2, scopolamine and atropine). *^b^* Medium spiked level (3 mg/kg), *^c^* High spiked level (6.25 mg/kg).

**Table 3:** The concentration of opium and tropane alkaloids in breadstick samples 1–4 ^a^.

Analyte	Breadsticks 1		Breadsticks 2		Breadsticks 3		Breadsticks 4	
	mg/100 g d.w.	Degradation (%)	mg/100 g d.w.	Degradation (%)	mg/100 g d.w.	Degradation (%)	mg/100 g d.w.	Degradation (%)
Mor ^b^	0.012–0.014	0–11	0.020–0.026	0–27	0.016–0.036	0–27	0.025–0.031	0–12
Atro ^c^	0.12 ± 0.01	20 ± 6	0.14 ± 0.01	7 ± 4	0.51 ± 0.02	64 ± 1	0.51 ± 0.02	65 ± 1

^a^ Sample preparation details in Figure 1 and Table 1. ^b^ Mor: morphine; ^c^ Atro: atropine. Results as mean (mg/100 g dry weight, d.w.) ± standard deviation (SD) of six replicates, except for morphine where a range is shown due to natural variation in the seeds.

**Table 4:** The concentration of opium and tropane alkaloids in breadstick samples 5–7 ^a^.

Analyte	Breadsticks 5		Breadsticks 6		Breadsticks 7	
	mg/100 g d.w.	Degradation (%)	mg/100 g d.w.	Degradation (%)	mg/100 g d.w.	Degradation (%)
Morphine	ND ^b^	100	0.003–0.006	0–81	0.003–0.007	0–77
Codeine	ND	100	0.0001 ± 0.0005	>88	0.004 ± 0.001	>88
Thebaine	0.004 ± 0.002	>58	0.004 ± 0.002	>58	0.003 ± 0.001	>58
Papaverine	0.020 ± 0.001	55 ± 2	0.0209 ± 0.0004	53 ± 1	0.023 ± 0.0004	49 ± 1
Noscapine	0.035 ± 0.001	23 ± 2	0.034 ± 0.003	24 ± 7	0.039 ± 0.001	14 ± 1
Atropine	0.093 ± 0.002	35 ± 1	0.09 ± 0.01	34 ± 9	0.11 ± 0.02	32 ± 4
Scopolamine	NP ^c^	NP	NP	NP	0.08 ± 0.01	45 ± 6
Anisodamine-1	NP	NP	NP	NP	0.14 ± 0.05	35 ± 20
Anisodamine-2	NP	NP	NP	NP	0.17 ± 0.08	49 ± 15

^a^ Sample preparation details in Figure 1 and Table 1. ^b^ ND: not detected. ^c^ NP: not added to the sample. Results as mean (mg/100 g dry weight, d.w.) ± standard deviation (SD) of 6 replicates, except for morphine where a range is shown due to natural variation in the seeds.

## Data Availability

Data is contained within the article or supplementary material.

## Supplementary Materials

The following supporting information can be downloaded at: https://www.mdpi.com/article/10.3390/foods11152196/s1, Figure S1: Chemical structures of opium (morphine, codeine, thebaine, noscapine and papaverine) and tropane (scopolamine, atropine and anisodamine) alkaloids analysed; Figure S2: Chromatographic separation of tropane and opium alkaloids at their wavelength of maximum absorption under the optimised chromatographic conditions; Figure S3: (a) Optimisation of extraction solvent with 1 g of sample and (b) sample amount optimisation using methanol with 0.1% acetic acid as solvent extraction. Table S1: Mobile phase composition and gradient elution used in the HPLC-DAD separation; Table S2: Chromatographic parameters obtained in the separation by HPLC-DAD; Table S3: Instrumental validation parameters for HPLC-DAD analysis; Table S4: Range of opium alkaloids in poppy seeds found in the literature [14,20,21,38,39,40]; Table S5: Concentration of tropane and opium alkaloids in breadsticks samples before and after baking process and percentage of degradation; Table S6: Summary of thermal processing leading to reduction in TAs and OAs content in cereal-based foods [20,21,22,23,40,47].
