# Supplementary material for: Evaluation of Thermal Degradation of Tropane and Opium Alkaloids in Gluten-Free Corn Breadsticks Samples Contaminated with Stramonium Seeds and Baked with Poppy Seeds under Different Conditions
Source: Foods. 2022 Jul 23;11(15):2196. doi: 10.3390/foods11152196 (PMC9330423; doi:10.3390/foods11152196)
Supplement: Supplementary file 1 [file foods-11-02196-s001.zip › foods-1761318-supplementary.pdf]

SUPLEMENTARY INFORMATION

Evaluation of thermal degradation of tropane and opium alkaloids in gluten-free corn breadsticks samples contaminated with stramonium seeds and baked with poppy seeds under different conditions

Fernando L. Vera-Baquero, Sonia Morante-Zarcero and Isabel Sierra*

Departamento de Tecnología Química y Ambiental, E.S.C.E.T., Universidad Rey Juan Carlos, C/Tulipán s/n, Móstoles, 28933 Madrid, Spain
fernando.vera@urjc.es (F.V.-B.); sonia.morante@urjc.es (S.M.-Z.)

* Correspondence: isabel.sierra@urjc.es (I.S.), Tel.: +34-91-488-70-18.

Table S1. Mobile phase composition and gradient elution used in the HPLC-DAD separation.

Time (min)	Solvent A (%)	Solvent B (%)	Flow rate (mL/min)
0.0	98	2	1.000
2.0	98	2	1.000
5.0	85	15	1.000
6.5	75	25	1.000
8.5	65	35	1.000
10.0	65	35	1.000
10.5	98	2	1.000
12.0	98	2	1.000

Solvent A: Water + 0.1% trifluoroacetic acid; Solvent B: Acetonitrile

Table S2. Chromatographic parameters obtained in the separation by HPLC-DAD.

Analytes	t_R	k	α	As	N	Rs	λ
Morphine	5.068	5.3	-	1.00	68211	-	212
Codeine	6.561	7.1	1.29	1.00	140884	20.0	212
Anisodamine-1	6.796	7.4	1.03	1.07	149239	3.3	212
Scopolamine	6.997	7.7	1.02	1.19	188446	2.9	212
Anisodamine-2	7.085	7.8	1.01	1.06	212570	1.3	212
Atropine	7.678	8.5	1.08	1.00	193638	9.2	212
Thebaine	8.486	9.5	-	1.00	210353	-	225
Papaverine	9.058	10.2	-	1.00	217833	-	240
Noscapine	9.192	10.4	1.19	1.00	219651	20.8	212

Chromatographic conditions: InfinityLab Poroshell 120 EC-C18 (3.0 x 150 mm, 2.7 µm) column; solvent A (water with 0.1% trifluoroacetic acid), solvent B (acetonitrile); elution gradient as Table S2, flow rate 1.000 mL/min; injection volume: 20 µL; column temperature: 30 °C; analysis time: 12 min.
t_R: retention time (min); k: capacity factor = $\frac{t_R - t_0}{t_0}$; α: selectivity = $\frac{k_b}{k_a}$; As: asymmetry factor = $\frac{a+b}{2a}$, where a + b are the width made at 10% of the maximum peak height, which is divided into two parts, left (a) and right (b), by the perpendicular drawn from the point of intersection of the tangents; N: theoretical plate number =$16\left(\frac{t_R}{w_B}\right)^2$; Rs: resolution = $\frac{2(t_{R(b)} - t_{R(a)})}{w_{B(b)} + w_{B(a)}}$; λ: wavelength (nm).

Table S3. Instrumental validation parameters for HPLC-DAD analysis.

Analytes	Linear range (mg/L)	Calibration line (R^2)	LOQ (mg/L)	LOD (mg/L)
Morphine	0.1-5	y = 329.07x – 3.8797 (0.9998)	0.10	0.03
Codeine	0.1-5	y = 409.05x + 2.9271 (0.9999)	0.10	0.03
Anisodamine-1	1-25	y = 68.357x - 4.2598 (0.9997)	0.52	0.16
Scopolamine	0.2-25	y = 78.697x + 9.4647 (0.9999)	0.20	0.07
Anisodamine-2	0.5-25	y = 46.055x + 2.308 (0.9999)	0.52	0.15
Atropine	0.4-25	y = 122.48x + 7.9366 (1)	0.40	0.12
Thebaine	0.1-5	y = 319.27x - 1.3183 (0.9996)	0.10	0.03
Papaverine	0.05-5	y = 632.3x + 1.8786 (0.9999)	0.01	0.004
Noscapine	0.075-4	y = 776.09x + 1.3524 (1)	0.06	0.02

LOQ: limits of quantification; LOD: limits of detection determined as 3 and 10 times the signal/noise ratio, respectively.

Table S4. Range of opium alkaloids in poppy seeds found in the literature.

Content (mg/Kg)					Reference
**Mor**	**Cod**	**The**	**Pap**	**Nos**	
4.1-22.3	<0.24-0.3	<2.40-1.5	<0.24	<0.24	[14]
0.2-241	<0.1-384	<0.1-106	<0.1-3.8	<0.1-5	[39]
ND-64	ND-23	ND-133	-	ND-11	[40]
<1-270	<1-56	-	<1	<1-2.1	[21]
3.6-261	1.9-378	8.1-217	-	-	[20]
<1-2788	<1-247	<1-124	-	-	[38]

Mor: morphine; Cod: codeine; The: thebaine; Pap: papaverine; Nos: noscapine.

ND: not detected; -: not analysed

Table S5. Concentration of tropane and opium alkaloids in breadsticks samples before and after baking process and percentage of degradation.

Sample	Analytes	Estimated concentration in breadsticks before baking (µg/g, w.w.)[a]	Expected concentration in breadsticks after baking (µg/g, d.w.)[b]	Found concentration in breadsticks after baking (µg/g, d.w.)[c]	Degradation ± SD (%)
**Breadsticks 1**	Morphine	0.006-0.103	0.009-0.140	0.125-0.143	0-11
	Atropine	1.06	1.43	1.20±0.14	20±6
**Breadsticks 2**	Morphine	0.013-0.207	0.017-0.279	0.205-0.265	0-27
	Atropine	1.06	1.43	1.35±0.07	7±4
**Breadsticks 3**	Morphine	0.006-0.103	0.009-0.140	0.164-0.358	0-27
	Atropine	10.62	14.35	5.20±0.21	64±1
**Breadsticks 4**	Morphine	0.013-0.207	0.017-0.279	0.247-0.350	0-12
	Atropine	10.62	14.35	5.08±0.15	65±1
**Breadsticks 5**	Morphine	0.006-0.103	0.009-0.140	0	100
	Codeine	0.33	0.45	0	100
	Thebaine	0.33	0.45	MQL (0.18)	> 58
	Papaverine	0.33	0.45	0.20±0.01	55±2
	Noscapine	0.33	0.45	0.35±0.01	23±2
	Atropine	1.06	1.43	0.93±0.02	35±1
**Breadsticks 6**	Morphine	0.006-0.103	0.009-0.140	0.027-0.059	0-81
	Codeine	0.33	0.45	MQL (0.053)	> 88
	Thebaine	0.33	0.45	MQL (0.18)	> 58
	Papaverine	0.33	0.45	0.21±0.01	53±1
	Noscapine	0.33	0.45	0.37±0.01	24±7
	Atropine	1.06	1.43	0.94±0.13	34±9
**Breadsticks 7**	Morphine	0.006-0.103	0.009-0.140	0.032-0.070	0-77
	Codeine	0.33	0.45	MQL (0.053)	> 88
	Thebaine	0.33	0.45	MQL (0.18)	> 58
	Papaverinea	0.33	0.45	0.230±0.004	49±1
	Noscapinea	0.33	0.45	0.390±0.008	14±1
	Atropinea	1.06	1.43	1.1±0.2	32±4
	Scopolamine	1.06	1.43	0,8±0.1	45±6
	Anisodamine-1	1.06	1.43	1.3±0.5	35±20
	Anisodamine-2	1.06	1.43	1.6±0.8	49±15

[a] Morphine and atropine wet weight (w.w.) concentration in the non-baked breadsticks was estimated taking into account the concentration of these alkaloids in the poppy seeds (0.23-3.72 mg/kg of morphine) and in the stramonium seed powder (1911 ± 283 mg/kg of atropine) determinate in this work.

[b] Expected dry weight (d.w) concentration in the non-baked breadsticks. The weight loss after baking was 26 ± 1% (n = 12).

[c] Concentration (d.w) found in the analysed baked breadsticks.

Table S6. Summary of thermal processing leading to reduction in tropane and opium alkaloids content in cereal-based foods.

	Reduction (%)								
	**Atropine**	**Scopolamine**	**Anisodamine**	**Morphine**	**Codeine**	**Thebaine**	**Papaverine**	**Noscapine**	**Reference**
Bread (proofing)	34	54	35	-	-	-	-	-	[22]
Bread (baking)	73	84	83	-	-	-	-	-	[22]
Pasta (boiling)	82	86	86	-	-	-	-	-	[23]
Poppy cakes (baking)	-	-	-	50-84	50-90	-	-	-	[21]
Poppy buns (baking topping)	-	-	-	97	93	-	-	-	[21]
Muffin (baking)	-	-	-	0	0	0	-	-	[20]
Muffins (baking)	-	-	-	100	100	100	-	100	[40]
Poppy rolls (baking topping)	-	-	-	100	100	-	100	100	[40]
Cake (baking)	-	-	-	55-75	-	-	-	-	[47]

- Not determinated

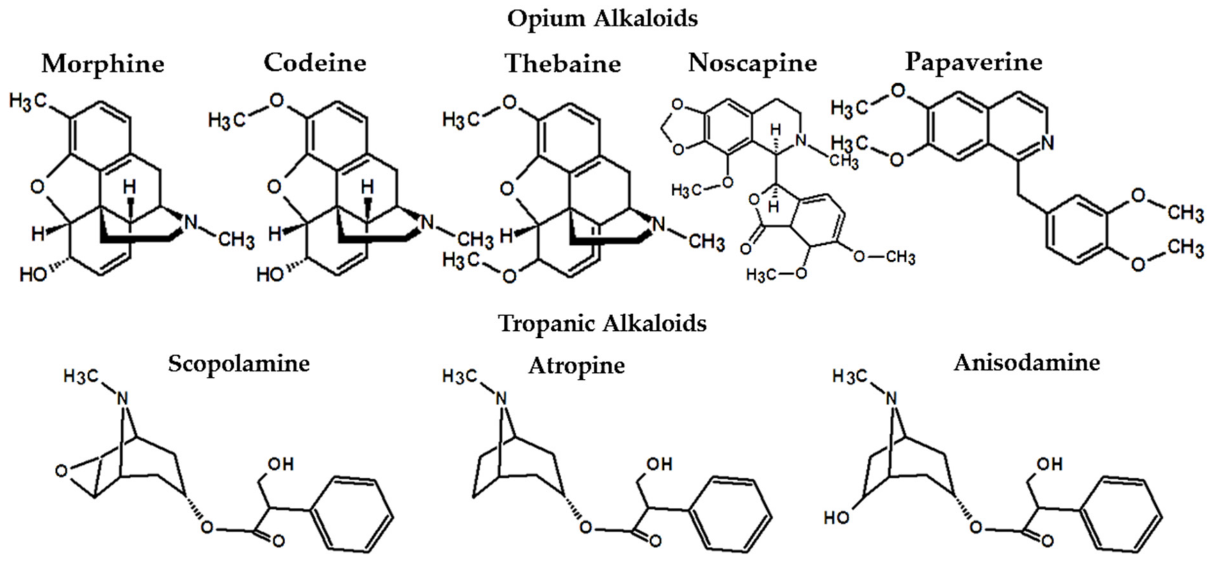

Figure S1. Chemical structures of opium (morphine, codeine, thebaine, noscapine and papaverine) and tropane (scopolamine, atropine and anisodamine) alkaloids analysed.

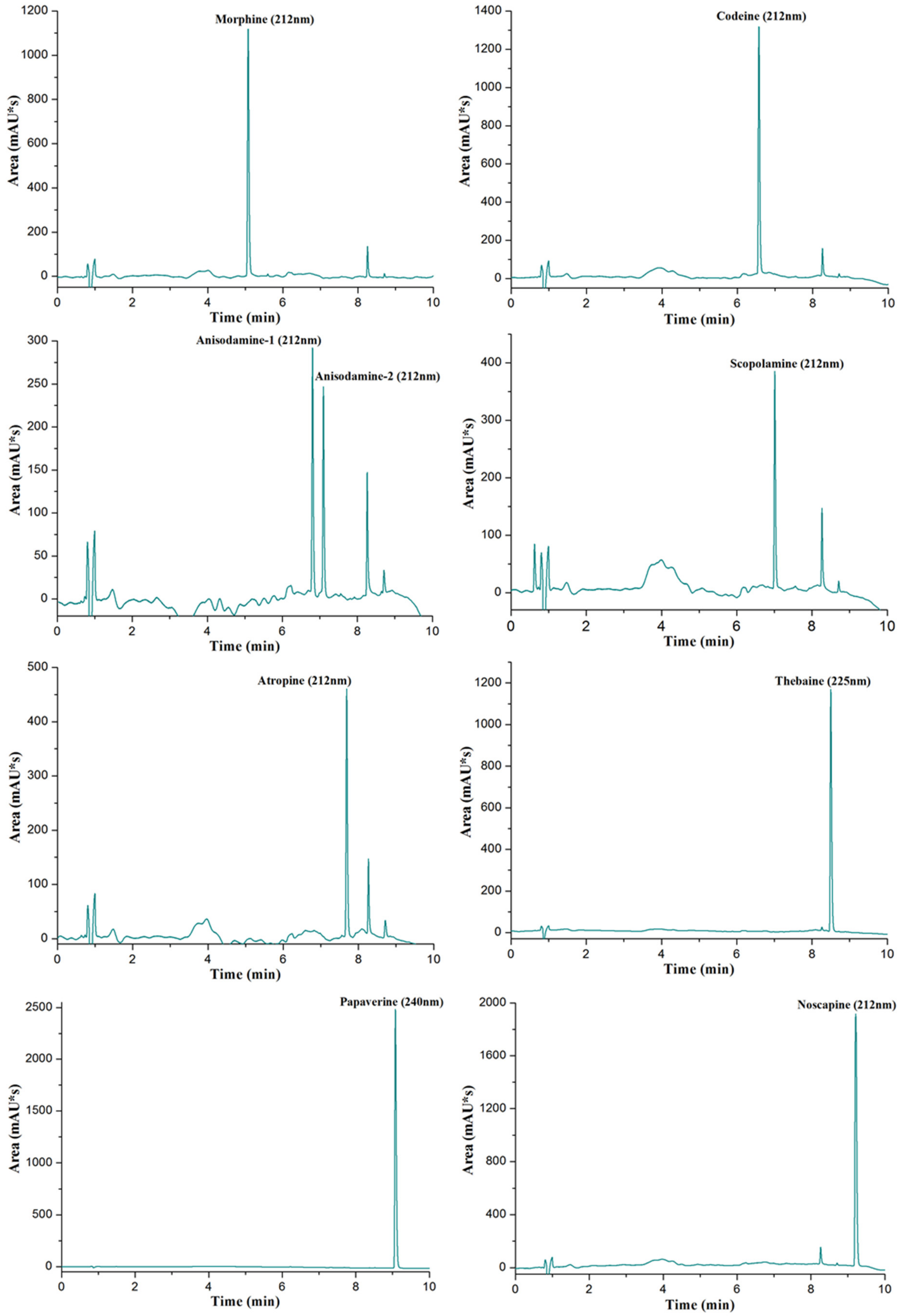

Figure S2. Chromatographic separation of tropane and opium alkaloids at their wavelength of maximum absorption under the optimised chromatographic conditions.

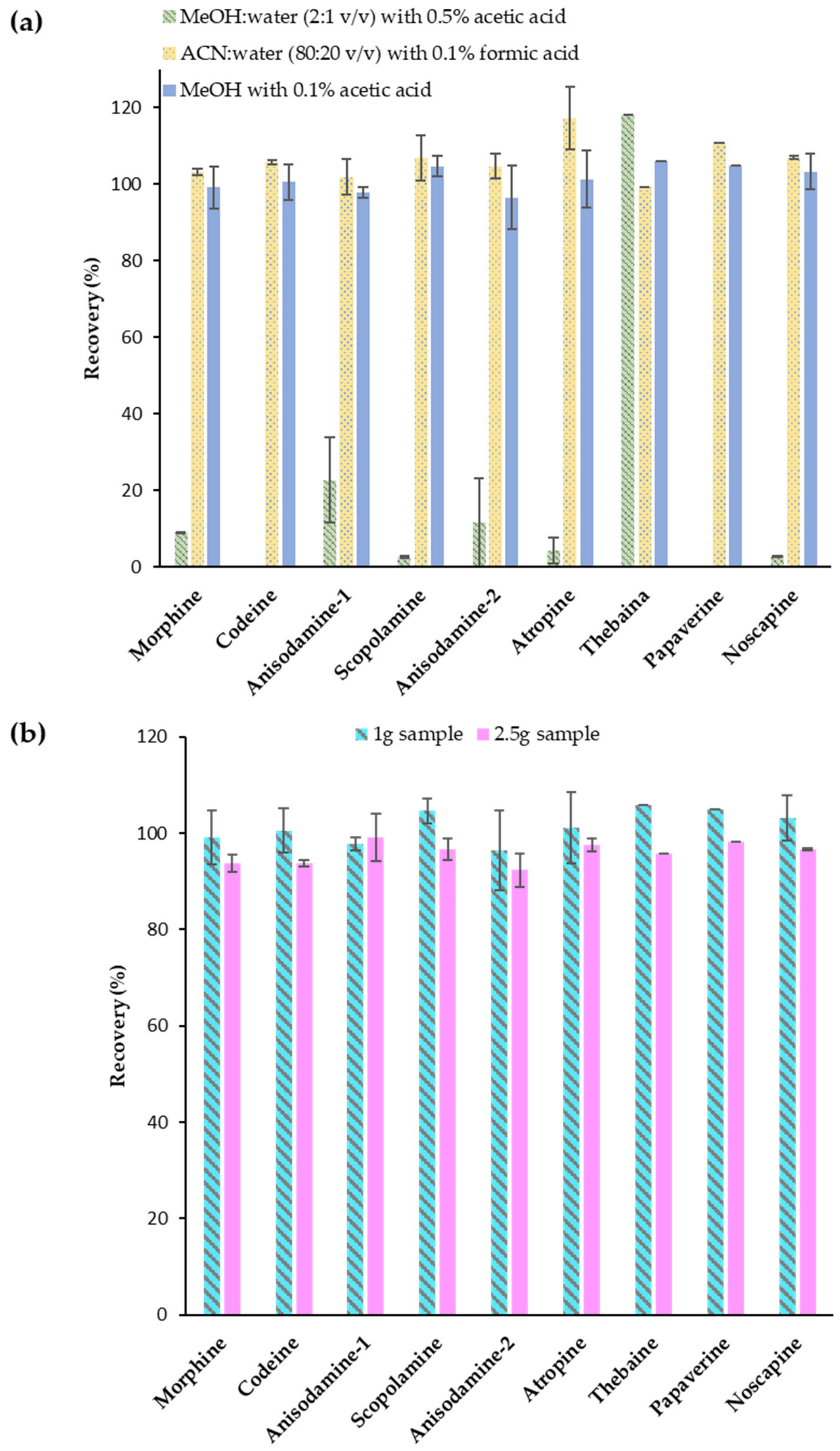

Figure S3. (a) Optimisation of extraction solvent with 1 g of sample and (b) sample amount optimisation using methanol with 0.1% acetic acid as solvent extraction.
